# Pharmacological Effect of GABA Analogues on GABA-ρ2 Receptors and Their Subtype Selectivity

**DOI:** 10.3390/life12010127

**Published:** 2022-01-17

**Authors:** Moawiah M. Naffaa, David E. Hibbs, Mary Chebib, Jane R. Hanrahan

**Affiliations:** School of Pharmacy, Faculty of Medicine and Health, University of Sydney, Sydney, NSW 2006, Australia; moawiah.naffaa@duke.edu (M.M.N.); david.hibbs@sydney.edu.au (D.E.H.); mary.collins@sydney.edu.au (M.C.)

**Keywords:** ionotropic GABA_A_ receptor, GABA-ρ2 receptors, orthosteric ‘GABA’ binding site, GABA analogues, additive/inhibitive effects, GABA-ρ subtype-selectivity

## Abstract

GABAρ receptors are distinctive GABAergic receptors from other ionotropic GABA_A_ and metabotropic GABA_B_ receptors in their pharmacological, biochemical, and electrophysiological properties. Although GABA-ρ1 receptors are the most studied in this subfamily, GABA-ρ2 receptors are widely distributed in the brain and are considered a potential target for treating neurological disorders such as stroke. The structure of GABA-ρ2 receptors and their pharmacological features are poorly studied. We generated the first homology model of GABA-ρ2 channel, which predicts similar major interactions of GABA with the binding-site residues in GABA-ρ1 and GABA-ρ2 channels. We also investigated the pharmacological properties of several GABA analogues on the activity of GABA-ρ2 receptors. In comparison to their pharmacological effect on GABA-ρ1 receptors, the activation effect of these ligands and their potentiation/inhibition impact on GABA response have interestingly shown inter-selectivity between the two GABA-ρ receptors. Our results suggest that several GABA analogues can be used as research tools to study the distinctive physiology of GABA-ρ1 and GABA-ρ2 receptors. Furthermore, their partial agonist effect may hold promise for the future discovery of selective modulatory agents on GABA_A_ receptors.

## 1. Introduction

In mammalian brains, the neurotransmitter GABA activates GABA-ρ receptors (also known as GABA_C_ or GABA_A-ρ_ receptors), which have distinctive structure and pharmacological properties [1]. GABA-ρ receptors (i.e., GABA-ρ1 and GABA-ρ2) form functional homomeric ion channels compared to the heteromeric GABA_A_ ion channels such as α1β2γ2 GABA_A_ receptors [2]. The pharmacological profile of GABA-ρ receptors is distinguished from GABA_A_ receptor subfamilies by their responses to several ligands. For instance, the partially folded GABA analogue, CACA (*cis*-aminocrotonic acid), is a partial agonist at GABA-ρ receptors and has no effect on GABA_A_ receptors [3]. Furthermore, TPMPA (1,2,5,6-tetrahydropyridin-4-yl) methylphosphinic acid) is a selective and a potent antagonist at GABA-ρ receptors [4]. On the other hand, GABA_A_ receptors can be modulated by agents such as barbiturates, benzodiazepines, and neurosteroids, while GABA-ρ receptors have no response to these modulators [5].

However, GABA-ρ subunits are highly similar in their structure; each subunit has distinctive features due to slight differences in the amino acid sequence [1]. For example, GABA-ρ1 and GABA-ρ2 subunits have residues different to the GABA_A_ subunits at two sites within the transmembrane 2 (TM2) region of the pore. Ligands that bind in the pore of GABA-ρ channels such as picrotoxin form several interactions with these two residues, which are essential for their selectivity to GABA-ρ channels [6,7]. On the other hand, ligands such as imidazole-4-acetic acid (I4 AA) that bind in the GABA binding sites can also show variable selectivity to the different GABA-ρ channels [8,9,10].

GABA-ρ2 subunits are widely distributed in brain tissues such as the hippocampus, cerebellum, and pituitary [11]. Several studies have suggested the involvement of GABA-ρ receptors in functions related to memory and learning [12], sleep-waking behaviour [13], hormone release in the pituitary [14], and inhibition of ammonia-induced apoptosis in hippocampal neurons [15]. They may also have functions in paired-pulse depression (a common form of short-term synaptic plasticity) of inhibitory postsynaptic currents [16]. A behavioural pharmacological study showed different in vivo functions of GABA-ρ receptors in the motor effect of ethanol [17].

A recent study has shown that GABA-ρ2 receptors can be a potential pharmacological target for ischemic stroke recovery [18]. The structural and pharmacological features of GABA-ρ2 receptors are still poorly understood. To reveal the unique properties of GABA-ρ2 receptors, we first generated a GABA-ρ2 homology model based on a well-validated GABA-ρ1 model in the open conformation. To our knowledge, this is the first model of the GABA-ρ2 channel, which was then used to predict the binding modes of GABA analogues in the orthosteric ‘GABA’ binding site. We subsequently implemented two-electrode voltage-clamp electrophysiology (TEVC) to record the pharmacological effect of the ligands in this study at GABA-ρ2 receptors expressed on the membrane of Xenopus oocytes. The GABA analogues used in this work were chosen because they have previously shown interesting pharmacological profiles at GABA-ρ1 WT (wild type) and mutant receptors [19,20]. In these studies, they showed variable partial agonistic effects, which helped to understand the role of several residues in channel stabilization and activation. We hypothesize that these ligands elicit distinctive and selective pharmacological properties at GABA-ρ2 receptors. Our docking studies in the GABA binding site of GABA-ρ2 model predicted that each ligand has different binding modes and affinities compared to GABA. The GABA analogues in this study also elicited unique partial agonistic effects at the GABA-ρ2 receptors. Surprisingly, these ligands show distinctive pharmacological characteristics at GABA-ρ2 receptors compared to their responses at GABA-ρ1 receptors. Such ligands can be used to study the unique physiology of GABA-ρ2 receptors and their involvement in neurological disorders such as stroke. Our aim in this study is also to shed light on the importance of understanding the mechanism of partial agonistic effect of GABA analogues on GABA_A_ receptors, which may hold promise for a drug discovery to selectively modulate the activity of functional GABA_A_ receptors [20].

## 2. Materials and Methods

### 2.1. Molecular Homology Modelling and Protein-Ligand Docking

#### 2.1.1. Homology Modelling of Human GABA-ρ2 Channel

The homology modelling methodology used to generate the model of human GABA-ρ2 channel was the same as previously reported in detail [21]. Due to the high percentage of amino acid identity and similarity between human GABA-ρ1 and human GABA-ρ2 genes, we used our homology model of human GABA-ρ1 to generate a homology model of GABA-ρ2 channels. Briefly, the protein sequences of human GABA-ρ2 gene were obtained from the universal protein resources (https://www.uniprot.org/uniprot/P28476) accessed on 15 September 2021 [22]. The sequence amino acid alignments were performed using the CLUSTALW program [23], and the manual adjustments were performed on specific regions of the sequence alignment using Schrödinger’s Prime 3.2 program [24]. Models of GABA-bound GABA-ρ2 were built based on the GABA-ρ1 model in an open conformation using Prime 3.2 software [24]. These generated models were reviewed and verified as described in our previous published work [19].

#### 2.1.2. Ligand and Protein Preparation

**Figure 1:**
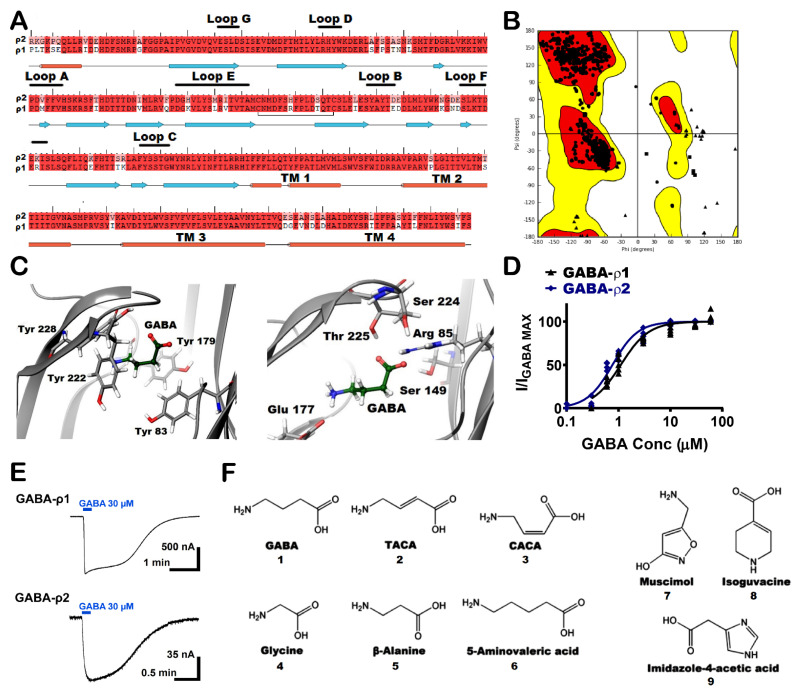
Molecular homology modelling of GABA-ρ2 receptors and chemical structure of used ligands in this study. (**A**) The alignment of the amino acid sequence of GABA-ρ1 and GABA-ρ2 subunits. The secondary structure was predicted by maestro according to the GABA-ρ2 model. (**B**) Ramachandran plots of the GABA-ρ2 homology model based on the GABA-ρ1 model (Glycine is plotted as triangles, proline is plotted as squares, all other residues are plotted as circles). (**C**) GABA docking results with GABA-ρ2 homology model based on a published GABA-ρ1 model in the open state, showing GABA surrounded by aromatic residues of the binding site (left image), and GABA surrounded by aliphatic residues of the binding site (right image). (**D**) The dose–response curves of GABA at GABA-ρ1 and GABA-ρ2 homomeric receptors (Mean ± S.E.M., *n* = 5). (**E**) Samples of GABA maximal response traces (30 μM) at GABA-ρ1 and GABA-ρ2 receptors. (**F**) Chemical structures of the tested ligands in this study.

The in silico ligands in this study were first generated using the 2D sketcher and then modified for chemical correctness using Schrödinger’s Maestro (Maestro, v9.9, Schrödinger, New York, NY, USA) interface (Figure 1E). All ligands were prepared for docking using LigPrep (LigPrep v3.1, Schrödinger, New York, NY, USA) [25]. Then, geometry minimizations were made on all conformations of ligands using the OPLS_2005 (MacroModel, v10.5) force field and the Truncated Newton Conjugate Gradient (TNCG) method [26]. Optimizations were converged to a gradient RMSD below 0.05 kJ/mol or continued to a maximum of 5000 iterations, at which there were insignificant changes in RMSD gradients.

Schrödinger’s protein preparation wizard was used to prepare the model of human GABA-ρ2 channel by adding hydrogens, adjusting bond orders and formal charges, and reducing potential steric clashes via minimizing the channel with the OPLS_2005 force field. The produced template was shown to be accurate structures without require for manual adjustment.

#### 2.1.3. Docking of Ligands into the GABA Binding Site of GABA-ρ2 Model

The default settings (i.e., flexible ligand sampling and added Epik state penalties to the docking score) of the Glide program implemented in Schrödinger Suite 20 was first used to perform the docking studies of all studied ligands with the generated GABA-ρ2 model [21,27]. Ligands were then induced fit docked to study the interactions between ligands and residues in the binding site [6,28].

### 2.2. Molecular Biology

#### 2.2.1. Material

The subcloned human GABA-ρ1 cDNA into pcDNA1.1 (Invitrogen, San Diego, CA, USA) was generously donated by Dr. George Uh1 (National Institute for Drug Abuse, Baltimore, MD, USA). The subcloned human GABA-ρ2 cDNA into PKS (Invitrogen) was generously provided by Dr. Garry Cutting (Center for Medical Genetics, Johns Hopkins University School of Medicine, Baltimore, MD, USA).

GABA, muscimol, β-alanine, 5-amino valeric acid, glycine, isoguvacine, and imidazole-4-acetic acid were purchased from Sigma-Aldrich (Sigma-Aldrich Pty Ltd., Castle Hill, NSW 1765, Australia). TACA and CACA were prepared as previously reported [3].

#### 2.2.2. Transformation and Preparation of Plasmids

The plasmid DNAs of GABA-ρ1 and GABA-ρ2 subunits were transferred into TOP10 Chemically Competent *E. coli* (Invitrogen™, Thermo Fisher Scientific, Waltham, MA, USA). The plasmid DNAs were extracted after bacterial culture, harvesting, and lysis of the bacteria. DNAs were then Purified using Qiagen Spin Miniprep Kit (Qiagen, VIC, Australia). Agarose gel electrophoresis (0.9%) were run using the GelDoc 1000 (Bio-Rad Laboratories, Hercules, CA, USA) to test the quality of plasmid DNAs.

#### 2.2.3. DNA Sequencing

To confirm the plasmid DNAs, the sequence of the whole gene using specific primers was performed by the Australian Genome Research Facility Ltd. (AGRF, Westmead Millennium Institute, Westmead, Australia).

#### 2.2.4. In Vitro Transcription (cRNA Synthesis)

The plasmid DNAs of GABA-ρ1 and GABA-ρ2 were linearized using Xba1 and EcoRV restriction enzymes, respectively. The linearized plasmids DNA were purified by QIAquick PCR purification Kit (Qiagen, VIC, Australia). GABA-ρ1 and GABA-ρ2 cRNA were synthesized from linearized plasmid DNA using the T7 Transcription mMESSAGE mMACHINE Kit (Ambion, Austin, TX, USA).

Nanodrop Spectrophotometer (Thermo Scientific, Wilmington, DE, USA) were used to test for quantity and quality of the synthesized cRNA. Then, agarose gel electrophoresis was also run using the GelDoc 1000 (Bio-Rad Laboratories, Hercules, CA, USA) to test the purity of synthesized cRNA.

### 2.3. Electrophysiology

#### 2.3.1. Xenopus Oocytes Preparation and Injection

Ovary lobes were dissected from female *Xenopus laevis* (South Africa clawed frogs), which were removed as reported previously [19,29], in accordance with the National Health and Medical Research Council (NHMRC) of Australia’s ethical guidelines. This procedure is also approved by the University of Sydney’s animal ethics committee (2013/5915). In brief, the females of *Xenopus laevis* were anaesthetized using 0.17% 3-aminobenzoic acid ethyl ester with 0.02% NaCl for 10–15 min, and a lobe of the ovary was dissected. The lobes were washed with oocyte releasing buffer 2 (OR2: 82.5 mM NaCl, 5 mM HEPES, 2 mM KCl, 1 mM MgCl_2_.6H_2_O pH 7.5) and incubated with collagenase A (2 mg·mL^−1^ in OR2, Boehringer Mannheim, Mannheim, Germany) for approximately 2 h to separate oocytes from follicular cells and connective tissue. The released oocytes were washed in ND96 solution (96 mM NaCl, 1 mM MgCl2.6H2O, 2 mM KCl, 5 mM HEPES, and 1.8 mM CaCl2 pH 7.5). Stage V–VI oocytes were collected and injected in the cytoplasm using a 15–20 μm diameter tip micropipette (micropipette puller, Sutter Instruments, Novato, CA, USA) and a Nanoject injector (Drummond Scientific Co., Broomall, PA, USA) set to deliver a total of ~15 ng of cRNA.

The injected oocytes were placed on an oscillator at 18 °C for 2–6 days in ND96 solution, which contained 0.5 mM theophylline, 2.5 mM pyruvate, 50 μg/mL tetracycline, and 50 μg/mL gentamycin.

#### 2.3.2. Two-Electrode Voltage-Clamp Electrophysiology (TEVC)

The activity of receptors was tested by recording whole cell currents using the two-electrode voltage clamp technique as previously reported [19,29]. In brief, fabricated microelectrodes prepared with a micropipette puller (Sutter instruments, Novato, CA, USA), were filled with 3 M KCl solution (0.3–2.3 MΩ). Oocytes were immersed with ND96 buffer in the cell chamber at approximately 5 mL/min rate. The microelectrode impaled cells were clamped at −60 mV (voltage), and the currents elicited in response to the application of ligand/s was recorded using a Power Lab 2/25 analogue–digital data acquisition system (AD Instruments, Sydney, NSW, Australia), a Geneclamp 500 B amplifier (Axon Instrument, Foster City, CA, USA), and Lab Chart version 5.0.2 program (ADInstruments, Sydney, Australia).

The stock solutions of GABA, TACA, CACA, glycine, β-alanine, and muscimol were prepared in distilled water, while the rest of ligands were prepared in dimethyl sulfoxide (DMSO). Ligands were applied into the perfusate until a peak response (maximal) was reached. All stock solutions of the ligands were freshly made on the day of experiments. Solutions of ligands dissolved in DMSO were standardized to contain less than 0.8% DMSO in the final perfusate solution to prevent any alteration in the recordings. Perfusates containing these ligands (chemicals) were freshly prepared before each application. The dose–response curves of ligands were obtained by increasing the concentrations of applied agonists on oocytes until the maximum current reached. The wash out periods ranged from 3.5 to 15 min depending on the solubility and concentration of the applied ligand on oocytes to allow for receptors to recover from desensitization. All experiments were conducted using at least two batches of oocytes.

#### 2.3.3. Data Analysis and Statistical Procedures

All current responses were normalized to the GABA maximal recorded current in the same cell and expressed as a percentage, which was fitted by least squares to Hill equation (Equation (1)). The GraphPad PRISM 8.02 was used to generate the dose–response curves for GABA and other ligands (GraphPad Software, San Diego, CA, USA). The responses of tested ligands at GABA-ρ1 and GABA-ρ2 receptors were normalized by GABA EC_max_ concentrations to generate their dose–response curves.

I = I_max_ [A]^nH^ / (EC_50_^nH^ + [A]^nH^) (1)

where I is the current response, referring to a known concentration of agonist; I_max_ is the maximum current obtained; [A] is the agonist concentration; EC_50_ is the concentration of agonist/partial agonist at which the current response is half maximal; and nH is the Hill coefficient.

The inhibitory concentration curves were also constructed using GraphPad PRISM 8.02, and IC_50_ values were calculated using Equation (2)

I = I_max_ [A]^nH^ / (IC_50_^nH^ + [A]^nH^) (2)

where I is the peak current at a given concentration of agonist, I_max_ is the maximal current generated by the concentration of agonist, [A] is the concentration of GABA, IC_50_ is the antagonist concentration which inhibits 50% of the maximum GABA response; and nH is the Hill coefficient.

The potentiation and inhibition effects of co-application of glycine and β-alanine with GABA EC_50_ on GABA-ρ2 receptors were fitted to the bell-shaped and biphasic dose–response curves, respectively. The equations for bell-shaped and biphasic curves are embedded in GraphPad PRISM 8.02.

Student’s *t*-test was used to determine the statistical significance of the change in the potentiation/inhibition effects of GABA analogues at GABA-ρ2. A *p* value < 0.05 was considered statistically significant. Detailed statistical results are described in figures and their legends. Significance assigned * if *p* < 0.05, ** if *p* < 0.01, *** if *p* < 0.001 and **** if *p* < 0.0001.

## 3. Results

### 3.1. GABA-ρ2 Receptors: Homology Modeling and Activity of GABA

A group of GABA-ρ2 homology models was constructed based on a previously published GABA-ρ1 in the open conformation using GABA as the reference ligand [21]. After the sequence alignment, the proteins were found to be homologous with 88% amino acid sequence identity, 94% amino acid sequence similarity, and 0% gaps (Figure 1A). In this set of GABA-ρ2 models, the best five models were chosen according to both RMS derivative OPLS_2005 and potential energy OPLS_2005. The structure of the selected model was carefully examined, especially the residues in the orthosteric binding site region. The energetic properties and stereochemical along with the packing environment of the residues were checked by Ramachandran plot (Figure 1B). The selected model was also found to have good geometry properties with 97% of residues presented in the most favoured region and 99% in the allowed region. The residues, which were found in the generously allowed or disallowed regions, are not in the binding pocket or known for any critical role in the ligand binding or channel gating processes. The percentages of bad backbone bonds and bad backbone angles were found to be 0.12% and 0.2%, respectively. Although there have been no mutational studies conducted on the extracellular domain of the GABA-ρ2 subunits, their high amino acid homology with GABA-ρ1 subunits could promote the study of GABA binding site in the GABA-ρ2 model and the numerous interactions of GABA and other ligands with the residues of the binding site. Although the amino acid sequence alignment of the GABA binding site region of the GABA-ρ1 and GABA-ρ2 subunits revealed high homology, there are some non-conserved aliphatic hydrophobic residues in Loop E (Figure 1A).

The main interactions between GABA and the residues in the orthosteric binding site of GABA-ρ2 homology model were predicted to be similar to those found in the GABA-ρ1 channel. This was verified by the homology model showing that GABA forms two salt bridges with Arg83 and Glu177. The carboxylate group of GABA forms H-bonds with the aliphatic hydrophilic residues Ser149 and Thr225 (Figure 1C, right). In addition, the cationic ammonium group of GABA is surrounded by aromatic residues (i.e., Tyr83, Tyr179, Tyr222, and Tyr228) to stabilize ligands in the binding site by forming an aromatic box (Figure 1C, left).

GABA is slightly more potent at GABA-ρ2 receptors (EC_50_ = 800 nM) than at GABA-ρ1 receptors (EC_50_ = 1 µM) expressed in oocytes (Figure 1D); however, the maximum amplitude of GABA current response at GABA-ρ2 receptors is approximately 10-fold less than at GABA-ρ1 receptors (Figure 1E).

### 3.2. Docking of GABA Analogues into the GABA Binding Site of GABA-ρ2 Model

To further investigate the GABA binding site of GABA-ρ2 model, GABA analogues in this study were docked into the binding site (i.e., unsaturated analogues, TACA (2) and CACA (3); aliphatic analogues, glycine (4), β-alanine (5), and 5-amino valeric acid (6); and aromatic analogues, muscimol (7), isoguvacine (8), and imidazole-t4-acetic acid (9)) (Figure 1F). The docking studies were undertaken to study the binding modes of these GABA analogues and their various interactions with residues of the GABA binding site in the GABA-ρ2 model. This may facilitate our understanding of their pharmacological effects at GABA-ρ2 receptors. 

**Figure 2:**
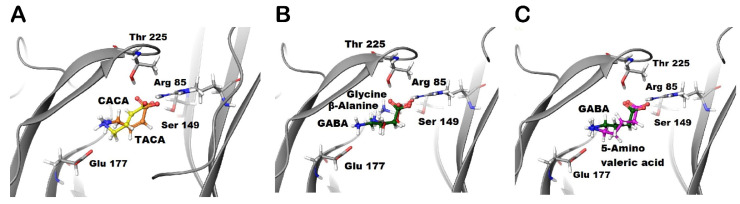
Docking of the studied aliphatic ligands into the GABA binding site of GABA-ρ2 model. Docking results of (**A**) TACA (orange) and CACA (yellow), (**B**) GABA (green) along with glycine (grey) and β-alanine (red), and (**C**) GABA (green) along with 5-amino valeric acid (purple) in the GABA binding site of GABA-ρ2 model in the open conformation.

The docking of TACA (orange) and CACA (yellow) predict a common interaction between these ligands and Arg85 residue. The ammonium groups of TACA and CACA are not close enough for Glu177 residue to form the salt bridge as with GABA’s ammonium group (Figure 2A). Although GABA was predicted to form H-bonds with both the Thr225 and Ser149 residues, TACA is predicted to form an H-bond with the Ser149 residue only. On the other hand, CACA is predicted to form an H-bond with the Thr225 residue only (Figure 1C and Figure 2A). Additionally, TACA and CACA may form different hydrophobic interactions with residues of loops D compared to GABA; therefore, the predicted conformational changes due to binding of TACA or CACA may stabilize binding sites in different ways during the closed and open states.

**Figure 3:**
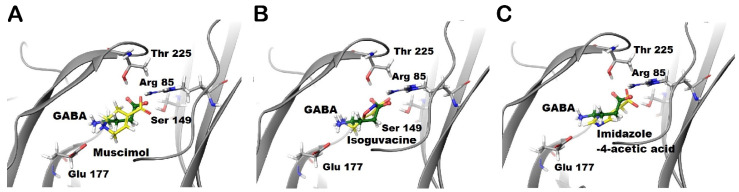
Docking of the studied aromatic ligands into the GABA binding site of GABA-ρ2 model. Docking results of GABA (green) along with (**A**) muscimol (yellow), (**B**) isoguvacine (yellow), and (**C**) I-4-AA (yellow) in the GABA binding site of GABA-ρ2 homology model in the open conformation.

The docking of glycine (grey) and β-alanine (red) predict that the carboxylate groups of these ligands form similar interactions with the receptor to GABA. The ammonium groups of glycine and β-alanine are not close to Glu177 residue, which suggests that these ligands are not able to form the salt bridge similar to GABA (Figure 2B). On the other hand, docking studies of 5-amino valeric acid (purple) predict that the negative and positive poles of this ligand (i.e., carboxylate and ammonium groups) form similar interactions to GABA. However, the extra methylene group in 5-amino valeric acid may provide additional contacts with residues in loops D, E, and F of the binding sites. These interactions may affect the ability of the 5-amino valeric acid to stabilize the channel in the closed and/or open states (Figure 3C).

The docking of muscimol (yellow) and isoguvacine (yellow) (Figure 3A,B, respectively) predict that muscimol forms similar interactions to GABA, whereas isoguvacine is predicted to form an additional cation-π interaction with Tyr228. Although the ammonium group of isoguvacine is not predicted to form a salt bridge with Glu177 residue, it is stabilized by an aromatic box of Tyr179, Tyr222, and Tyr228 residues (Figure 3B). On the other hand, imidazole-4-acetic acid (I-4-AA) (yellow) forms π-π stacking interactions with Phe119 and Tyr179 residues in the binding site (Figure 3C). While the imidazole ring in I-4-AA is predicted to form multiple stacking interactions with tyrosine residues, this ligand also forms a salt bridge with Arg85 only and a H-bond with Ser149 (Figure 3C).

Interestingly, the docking results predicted variously different binding modes for the studied GABA analogues into the GABA binding site of GABA-ρ2 model. We then investigated the pharmacological effect (i.e., potency, efficacy, and additive/inhibition effects) of these GABA analogues at human GABA-ρ2 receptors expressed in oocytes.

### 3.3. Pharmacological Effect of TACA and CACA at GABA-ρ2 Receptors

**Figure 4:**
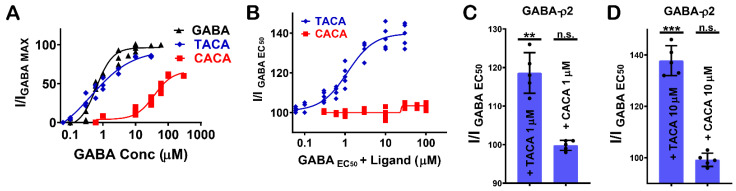
The pharmacological effect of TACA and CACA at GABA-ρ2 receptors. (**A**) The dose–response curves of GABA, TACA and CACA at GABA-ρ2 receptors (Mean ± S.E.M., *n* = 5). (**B**) The dose–response curves of TACA and CACA co-applied with GABA EC_50_ at GABA-ρ2 receptors (Mean ± S.E.M., *n* = 5). (**C**) The efficacy of the co-application of 1 μM TACA or 1 μM CACA with GABA EC_50_ (800 nM) at GABA-ρ2 receptors; unpaired *t*-test. ** *p*-value = 0.0025 and *p*-value = n.s., respectively. (**D**) The efficacy of the co-application of 10 μM TACA or 10 μM CACA with GABA EC_50_ (800 nM) at GABA-ρ2 receptors; unpaired *t*-test. *** *p*-value = 0.0001 and *p*-value = n.s., respectively.

The GABA analogues with an unsaturated carbon–carbon bond (i.e., TACA (2) and CACA (3), Figure 1F) are partial agonists at GABA-ρ2 receptors with relative efficacies of 85% and 60% to GABA, respectively (Figure 4A). Although TACA is not a full agonist at GABA-ρ2 receptors, it is slightly more potent than GABA with an EC_50_ = 450 nM, whereas CACA is significantly less potent than GABA and TACA with an EC_50_ = 38 µM [10].

The co-administration of TACA and CACA with GABA EC_50_ (800 nM) at GABA-ρ2 receptors shows that TACA elicited an additive effect to the GABA response; however, CACA had no significant effect on GABA response (Figure 4B). The GABA EC_50_ response increases with higher concentrations of TACA from 1 µM to 10 µM (118.4% and 137.5 % of GABA EC_50_ when co-applied with 1 µM and 10 µM TACA, respectively) (Figure 4C,D). At 10 µM of TACA, the addition to GABA responses is comparable in magnitude to the response elicited by TACA alone. This may indicate that TACA is either activating the unoccupied channels or binding at vacant sites of receptors where GABA is also bound. In contrast to TACA, the co-application of GABA EC_50_ with 1 mM of CACA does not show any significant effect on the GABA responses (data not shown). This may indicate that at GABA-ρ2 receptors, CACA is unable to compete with GABA at binding sites and has no additive effect on the GABA responses by binding at vacant sites.

### 3.4. Pharmacological Effect of Glycine, β-Alanine, and 5-Amino Valeric Acid at GABA-ρ2 Receptors

**Figure 5:**
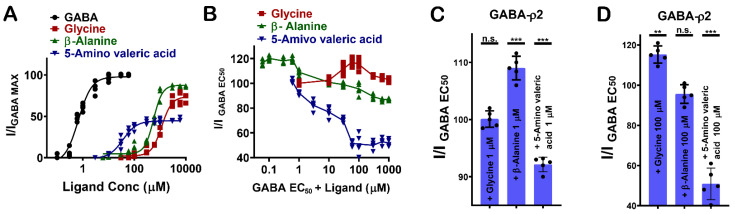
The pharmacological effect of glycine, β-alanine, and 5-amino valeric acid at GABA-ρ2 receptors. (**A**) The dose–response curves of GABA, glycine, β-alanine and 5-amino valeric acid at GABA-ρ2 receptors (Mean ± S.E.M., *n* = 5). (**B**) The dose–response curves of glycine, β-alanine and 5-amino valeric acid co-applied with GABA EC_50_ (800 nM) at GABA-ρ2 receptors (Mean ± S.E.M., *n* = 5). (**C**) The efficacy of the co-application of 1 μM glycine, 1 μM β-alanine and 1 μM 5-amino valeric acid with GABA EC_50_ (800 nM) at GABA-ρ2 receptors; unpaired *t*-test. *p*-value = n.s., *** *p*-value = 0.0003, and *** *p*-value = 0.0003, respectively. (**D**) The efficacy of the co-application of 100 μM glycine, 100 μM β-alanine and 100 μM 5-amino valeric acid with GABA EC_50_ (800 nM) at GABA-ρ2 receptors; unpaired *t*-test. ** *p*-value = 0.0016, *p*-value = n.s., and *** *p*-value = 0.0002, respectively.

GABA analogues with varying numbers of carbon atoms (i.e., glycine (4), β-alanine (5), and 5-amino valeric acid (6)) show distinctive pharmacological characteristics at GABA-ρ2 receptors (Figure 1F). 5-Amino valeric acid activates GABA-ρ2 receptors in a moderately potent and efficacious manner, while β-alanine and glycine activate the receptors with low potency but are highly efficacious compared to GABA (Figure 5A).

The co-application of glycine, β-alanine, and 5-amino valeric acid with GABA EC_50_ (800 nM) demonstrates different pharmacological effects at GABA-ρ2 receptors (Figure 5B). At 1 μM, glycine does not show a significant effect on GABA EC_50_ responses, but it has a very small additive effect at 100 μM (115.4% of GABA EC_50_ when co-applied with 100 µM glycine) (Figure 5C,D). While β-alanine has a weak additive effect on GABA EC_50_ responses at 1 μM, it has no significant effect on GABA EC_50_ responses at 100 μM (108.9% of GABA EC_50_ when co-applied with 1 µM β-alanine) (Figure 5C,D). 5-Amino valeric acid elicits a moderate antagonist effect on the GABA EC_50_ responses (92% and 51.1 % of GABA EC_50_ when co-applied with 1 µM and 100 µM 5-amino valeric acid, respectively) (Figure 5C,D). The activation efficacy of β-alanine and 5-amino valeric acid at GABA-ρ2 receptors is comparable to their inhibition efficacy of the GABA EC_50_ responses at the same receptors. This may suggest that carboxylate groups of GABA, glycine, β-alanine, and 5-amino valeric acid, which are predicted to form similar interactions in the binding site, are essential for the activation of the GABA-ρ2 channel.

### 3.5. Pharmacological Effect of Muscimol, Isoguvacine, and I-4-AA at GABA-ρ2 Receptors

**Figure 6:**
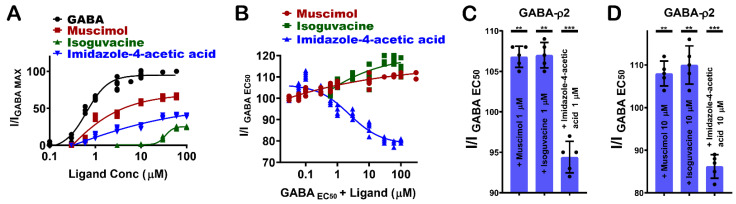
The pharmacological effect of muscimol, isoguvacine, and imidazole-4-acetic Acid (I-4-AA) at GABA-ρ2 receptors. (**A**) The dose–response curves of GABA, muscimol, isoguvacine, and I-4-AA at GABA-ρ2 receptors (Mean ± S.E.M., *n* = 5). (**B**) The dose–response curves of muscimol, isoguvacine and I-4-AA co-applied with GABA EC_50_ (800 nM) at GABA-ρ2 receptors (Mean ± S.E.M., *n* = 5). (**C**) The efficacy of the co-application of 1 μM muscimol, 1 μM isoguvacine, and 1 μM I-4-AA with GABA EC_50_ (800 nM) at GABA-ρ2 receptors; unpaired *t*-test. ** *p*-value = 0.0032, ** *p*-value = 0.0036, and *** *p*-value = 0.0007, respectively. (**D**) The efficacy of the co-application of 10 μM muscimol, 10 μM isoguvacine, and 10 μM I-4-AA with GABA EC_50_ (800 nM) at GABA-ρ2 receptors; unpaired *t*-test. ** *p*-value = 0.0043, ** *p*-value = 0.0066, and *** *p*-value = 0.0009, respectively.

GABA-ρ2 receptors show variable responses to the heterocyclic GABA analogues: muscimol (7), isoguvacine (8), and I-4-AA (9) (Figure 1F). Among these ligands, muscimol is the most potent and efficacious partial agonist [10], followed by I-4-AA [10] and isoguvacine, respectively (Figure 6A). The docking results may provide some explanations for the pharmacological features of muscimol at the GABA-ρ2 receptors. With similar interactions to GABA, muscimol has high efficacy; however, the heterocyclic carboxylate isostere of muscimol is not the most favourable for full agonist activity (Figure 3A).

The administration of muscimol with GABA EC_50_ (800 nM) shows a slight potentiation of the GABA responses at 1 µM and 10 µM muscimol (106.9 ± 1.1% and 107.9 ± 3.1% of GABA EC50 responses, respectively) (Figure 6B–D). Isoguvacine also elicited a small co-operative effect on GABA EC_50_ responses at concentrations below 100 µM (106.8 ± 2.5% and 110 ± 4.3% of GABA EC_50_ when co-applied with 1 µM and 10 µM isoguvacine, respectively) (Figure 6B–D). On the other hand, I-4-AA has an additive effect on GABA EC_50_ responses at nM ranges, and it weakly antagonizes GABA responses at 1 µM and 10 µM I-4-AA (94.2 ± 1.4% and 86.25 ± 3.5% of GABA EC_50_ responses, respectively) (Figure 6B–D). These results may suggest that muscimol and isoguvacine are not competing with GABA at the same binding sites of GABA-ρ2 receptors. However, they may bind to vacant sites to weakly potentiate the GABA response, whereas I-4-AA slightly competes with GABA on the same binding site of GABA-ρ2 receptors. This may confer the small antagonistic effect of the GABA EC_50_ responses at 1 µM and 10 µM I-4-AA (Figure 4D,E).

### 3.6. Inter-Subunit Selectivity between GABA-ρ1 and GABA-ρ2 Receptors

The pharmacological effects of the GABA analogues on GABA-ρ2 receptors were also compared with their activities on the GABA-ρ1 receptors. Interestingly, they elicit variable responses (i.e., potency and efficacy) and/or co-operative (additive)/inhibitive effects on GABA-ρ1 and GABA-ρ2 receptors (Table 1).

**Figure 7:**
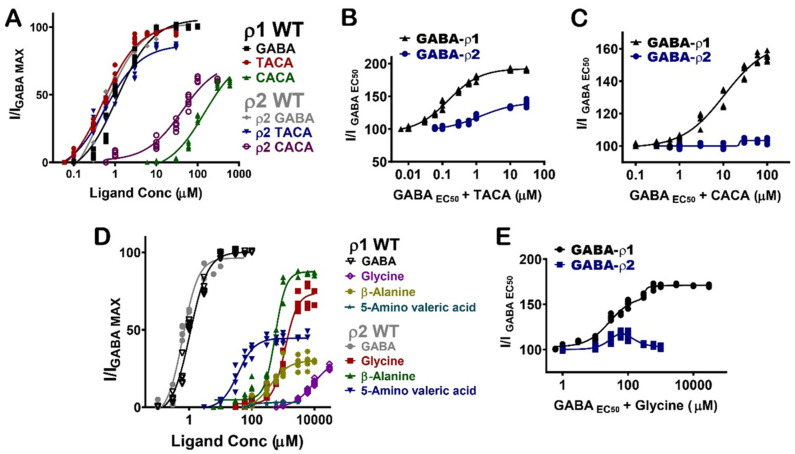
Pharmacological effects of GABA aliphatic analogues, and their inter-subunit selectivity at GABA-ρ1 and GABA-ρ2 receptors. (**A**) The dose–response curves of GABA, TACA, and CACA at GABA-ρ1 and GABA-ρ2 receptors (Mean ± S.E.M., *n* = 5). (**B**) The dose–response curves of the co-application of TACA with GABA EC_50_ (800 nM) at GABA-ρ1 and GABA-ρ2 receptors (Mean ± S.E.M., *n* = 5). (**C**) The dose–response curves of the co-application of CACA with GABA EC_50_ (800 nM) at GABA-ρ1 and GABA-ρ2 receptors (Mean ± S.E.M., *n* = 5). (**D**) The dose–response curves of GABA, glycine, β-alanine, and 5-amino valeric acid at GABA-ρ1 and GABA-ρ2 receptors (Mean ± S.E.M., *n* = 5). (**E**) The dose–response curves of the co-application of glycine with GABA EC_50_ (800 nM) at GABA-ρ1 and GABA-ρ2 receptors (Mean ± S.E.M., *n* = 5).

TACA and CACA have similar potency and efficacy at GABA-ρ1 and GABA-ρ2 receptors; however, their additive effects to GABA EC_50_ responses are significantly different (Figure 7A) (Table 1). While TACA shows 190% co-operative effect at GABA-ρ1 receptors, it shows only 140% co-operative effect at GABA-ρ2 receptors (Figure 7B). On the other hand, CACA shows 160% co-operative effects at GABA-ρ1 receptors and no significant effect (i.e., 104%) at GABA-ρ2 receptors (Figure 7C). This data suggests that TACA and CACA compete differently with GABA on binding sites of GABA-ρ1 compared to GABA-ρ2 receptors. Therefore, the overall responses are heterogeneous.

Glycine, β-alanine, and 5-amino valeric acid show significantly higher efficacy at GABA-ρ2 receptors than at GABA-ρ1 receptors (Figure 7D and Table 1). On another hand, β-Alanine has similar potency at GABA-ρ1 and GABA-ρ2 receptors, whereas glycine and 5-amino valeric acid are more potent at GABA-ρ2 receptors (Figure 7D and Table 1). In the presence of GABA, glycine has a 170% co-operative effect at GABA-ρ1 receptors, while it shows a little co-operative effect at GABA-ρ2 receptors (115% with 100 µM glycine) (Figure 7E and Table 1). β-Alanine shows a similar additive effect at both GABA-ρ receptor sub-types; however, its antagonistic effect is greater at GABA-ρ1 receptors (Table 1). While 5-amino valeric acid shows a slightly higher additive effect at GABA-ρ1 compared to GABA-ρ2, the inhibitory effect of 5-amino valeric acid is significantly higher at GABA-ρ1 receptors compared to its inhibition at GABA-ρ2 receptors (Table 1).

**Figure 8:**
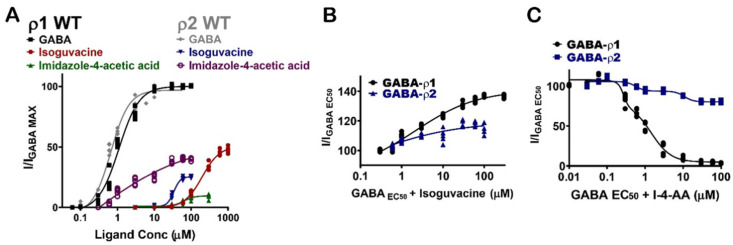
Pharmacological effects of GABA aromatic analogues, and their inter-subunit selectivity at GABA-ρ1 and GABA-ρ2 receptors. (**A**) The dose–response curves of GABA, isoguvacine, and I-4-AA at GABA-ρ1 and GABA-ρ2 receptors (Mean ± S.E.M., *n* = 5). (**B**) The dose–response curves of the co-application of isoguvacine with GABA EC_50_ (800 nM) at GABA-ρ1 and GABA-ρ2 receptors (Mean ± S.E.M., *n* = 5). (**C**) The dose–response curves of the co-application of I-4-AA with GABA EC_50_ (800 nM) at GABA-ρ1 and GABA-ρ2 receptors (Mean ± S.E.M., *n* = 5).

Muscimol shows similar potency and additive/inhibitive effects at both GABA-ρ1 and GABA-ρ2 receptors (Figure 8A) (Table 1). Although isoguvacine shows six-fold greater potency at GABA-ρ2 receptors, it has two-fold higher efficacy at GABA-ρ1 receptors (Figure 8A and Table 1). When isoguvacine is co-administrated with GABA EC_50_, it shows slightly higher additive effects at GABA-ρ1 receptors with no inhibition of the GABA response at either GABA-ρ1 or GABA-ρ2 receptors (Figure 8B and Table 1). I-4-AA shows significantly different potency and efficacy at GABA-ρ1 and GABA-ρ2 receptors. At GABA-ρ2 receptors, its potency and efficacy are 4- and 100-fold higher than at GABA-ρ1 receptors, respectively (Figure 8A) [8]. When I-4-AA is administered with GABA EC_50_, it shows a potent inhibitory effect at GABA-ρ1 receptors and a weak inhibitory effect at GABA-ρ2 receptors (Figure 8C and Table 1). These results suggest that I-4-AA has a unique binding mode and affinity to the GABA binding sites of each of GABA-ρ subtype receptors.

The docking (glide) scores of the docked GABA analogues into GABA-ρ2 model were compared with their scores in the GABA-ρ1 model. The docking results predicted that GABA analogues have nonsignificant binding affinities difference in the binding sites of GABA-ρ1 and GABA-ρ2 models (Table 2).

## 4. Discussion

### 4.1. Functional and Pharmacological Properties of GABA-ρ2 Receptors

Over the past three decades, it has been found that GABA-ρ2 receptors are widely distributed in the mammalian brain and are implicated in many neurological disorders [1]. Unlike GABA-ρ1 receptors, the structure and function of GABA-ρ2 receptors has not yet been explored or compared to that of GABA-ρ1 receptors. Our GABA-ρ2 homology model is the first generated structural model of the GABA-ρ2 channel. We used this model to understand the pharmacological effect of GABA analogues on the GABA-ρ2 receptors. TACA and CACA, which are GABA analogues with an unsaturated carbon–carbon bond, display different effects on GABA-ρ2 receptors. This may be explained by their binding mode in GABA-binding sites. With a similar binding mode to GABA, TACA has a similar effect on these channels and an additive effect to the GABA response when they are co-applied. With a different binding mode compared to GABA, CACA has moderate potency and efficacy and an insignificant effect on GABA responses. Further studies may look for additional residues in the binding site that are directly involved in the binding modes of these ligands and their responses by GABA-ρ2 receptors.

Among the hydrophilic analogues of GABA, 5-amino valeric acid, which has an extra carbon atom compared to GABA, is the most potent ligand at GABA-ρ2 receptors, while β-alanine, which has one less carbon atom compared to GABA, is the most efficacious. As predicted by docking studies in the binding site of GABA-ρ2 model, the missing amino group–salt bridge interactions of glycine and β-alanine may be not necessary for ligand efficacy at GABA-ρ2 receptors. On the other hand, 5-amino valeric acid, which is predicted to maintain all salt bridges in the binding site of GABA-ρ2 model, has low efficacy. Furthermore, the docking studies of these ligands predict various interactions with residues in Loops E and F, which may explain their different potency at GABA-ρ2 receptors. Future research is needed to reveal the functional roles of Glu177 residue and residues of loops E and F with side chains oriented toward the binding site, which may help to understand the distinctive potency and efficacy of these GABA analogues at GABA-ρ2 receptors.

The predicted interactions that heterocyclic GABA analogues muscimol, isoguvacine, and I-4-AA form in the binding site of GABA-ρ2 model may decipher their variable effects at GABA-ρ2 receptors. Muscimol, which is predicted to form similar interactions to GABA, has high potency and efficacy. On the other hand, isoguvacine, which is predicted to form a cation-π interaction instead of the salt bridge with Glu177 residue, has low efficacy and potency. The heterocyclic groups of these ligands are predicted to participate in several hydrophobic interactions with residues in loops D, E, and F. The small co-operative effects that muscimol and isoguvacine have on GABA responses may indicate that their hydrophobic interactions in the binding sites are not enough to replace GABA. I-4-AA elicits unique pharmacological activity at GABA-ρ2 receptors. While this ligand is predicted to form multiple pi-stacking interactions in the binding site, it shows moderate potency and efficacy. Furthermore, the weak antagonist effect of I-4-AA on GABA response indicates that this ligand slightly competes with GABA for binding sites. Future research to study the various predicted hydrophobic interactions of muscimol, isoguvacine, and I-4-AA in the binding site of GABA-ρ2 receptors may help to understand the unique function of these channels.

However, the docking scores of GABA analogues in the binding sites of GABA-ρ1 and ρ2 models show non-significant differences in their binding affinities, the slightly higher docking scores of these ligands in GABA-ρ1 model indicate that the non-conserved residues in loop E between the two GABA-ρ subunits may be result in the difference of their binding affinities and pharmacological effects between the two GABA-ρ receptors.

### 4.2. GABA Analogues: Tools to Explore the Unique Physiology of GABA-ρ Receptors and Future Therapeutic Options

The activation and additive/inhibitive effects of the studied GABA analogues revealed their selective pharmacological characteristics at GABA-ρ1 and GABA-ρ2 receptors. Based on the amino acid sequence of GABA-ρ1 and GABA-ρ2 subunits, loop E is a region where few residues are non-conserved between the two subunits. More studies to explore the role of these residues may explain the unique pharmacology of GABA-ρ1 and GABA-ρ2 receptors.

Several GABA analogues in this study such as CACA, glycine, 5-amino valeric acid, and I-4-AA show distinctive pharmacological activity between GABA-ρ1 and GABA-ρ2 receptors. These ligands can be used as research tools for future experiment to study the unique physiology and pathophysiology of GABA-ρ receptors. Generally, many GABA analogues are partial agonists on various GABA_A_ receptors with variable pharmacological properties based on their binding mode and affinity to GABA-binding sites. This partial agonistic effect may provide potential therapeutic options for neurological disorders.

## Figures and Tables

**Table 1:** Summary of the effects of GABA analogues at the GABA-ρ1 and GABA-ρ2 receptors (Potency, Efficacy and Additive/Inhibition effects).

Ligands.	GABA-ρ1 EC_50_	GABA-ρ1 Efficacy	GABA-ρ1 (Additive /Inhibition) (%)	GABA-ρ2 EC_50_	GABA-ρ2 Efficacy	GABA-ρ2 (Additive /Inhibition) (%)
TACA	EC_50_ = 0.6 ± 0.34 µM (Hill slope = 1.4 ± 0.16)	95%	190% (Hill slope = 0.9 ± 0.08)	0.45 ± 0.4 µM (Hill slope = 0.7 ± 0.54)	85%	140% (Hill slope = 0.8 ± 0.46)
CACA	93 ± 5.2 µM (Hill slope = 3.02 ± 0.6)	60%	160% (Hill slope = 0.9 ± 0.44)	37 ± 12 µM (Hill slope = 1.56 ± 0.61)	60%	104% (Hill slope =Not determined)
Glycine	≈1 mM (Hill slope = 1.35 ± 0.4)	25%	170% (Hill slope = 1.23 ± 0.42)	≈1 mM (Hill slope = 2.2 ± 0.78)	70%	(115/104) % (nH1 = 1.8 ± 0.3) (nH2 = 1.7 ± 0.5)
β-Alanine	400 µM (Hill slope = 1.45 ± 0.76)	30%	(120/40) % (Hill slope = 1.5 ± 0.8)	560 µM (Hill slope = 2.55 ± 0.62)	85%	(120/85) % (nH1 Not determined) (nH2 = 1.6 ± 0.36)
5-Aminovaleric acid	600 µM (Hill slope = Not determined)	3%	(125/2.5) % IC_50_ = 35 µM (Hill slope = 3 ± 0.75)	36 µM (Hill slope = 1.6 ± 0.55)	45%	(110/50) % IC_50_ = 10 µM (Hill slope = 0.6 ± 0.48)
Muscimol	1.25 µM	80%	125%	0.65 µM (Hill slope = 0.6 ± 0.62)	65%	112% (Hill slope = 0.14 ± 0.48)
Isoguvacine	205 µM (Hill slope = 1.82 ± 0.24)	40%	135% (Hill slope = 0.57 ± 0.25)	35 µM (Hill slope = 4.9 ± 1.9)	25%	(120/110) % (Hill slope = 0.37 ± 3.1)
Imidazole-4-acetic acid	66 µM (Hill slope = 3.1 ± 1.7)	10%	(115/3.5) % IC_50_ = 0.8 (Hill slope = 1.3 ± 0.22)	0.2 µM (Hill slope = 0.4 ± 0.35)	40%	(110/80) % IC_50_ = 2.4 µM (Hill slope = 0.87 ± 0.39)

**Table 2:** Summary of the glide (docking) score values of docked GABA analogues into the GABA binding sites of GABA-ρ1 and GABA-ρ2 models.

Ligands	GABA-ρ1 Glide Score (kcal/mol)	GABA-ρ2 Glide Score (kcal/mol)
GABA	−8.7	−8.3
TACA	−6.7	−5.2
CACA	−8.0	−7.9
Glycine	−5.5	−5.2
β-Alanine	−7.5	−6.5
5-Amino valeric acid	−9.3	−9.3
Muscimol	−10.4	−9.3
Isoguvacine	−8.4	−7.4
Imidazole-4-acetic acid	−7.6	−7.8

## Data Availability

Data is contained within the article.
